# A *yceI* Gene Involves in the Adaptation of *Ralstonia solanacearum* to Methyl Gallate and Other Stresses

**DOI:** 10.3390/microorganisms9091982

**Published:** 2021-09-17

**Authors:** Kai-Hao Wang, De-Hong Zheng, Gao-Qing Yuan, Wei Lin, Qi-Qin Li

**Affiliations:** College of Agriculture, Guangxi University, Nanning 530004, China; kyleofking@163.com (K.-H.W.); ygqtdc@sina.com (G.-Q.Y.); Linwei6419@126.com (W.L.)

**Keywords:** *Ralstonia solanacearum*, growth adaptability, methyl gallate

## Abstract

*Ralstonia solanacearum* is a plant-pathogenic bacterium causing plant bacterial wilt, and can be strongly inhibited by methyl gallate (MG). Our previous transcriptome sequencing of MG-treated *R. solanacearum* showed that the *yceI* gene *AVT05_RS03545* of Rs-T02 was up-regulated significantly under MG stress. In this study, a deletion mutant (named DM3545) and an over-expression strain (named OE3545) for *yceI* were constructed to confirm this hypothesis. No significant difference was observed among the growth of wild-type strain, DM3545 and OE3545 strains without MG treatment. Mutant DM3545 showed a lower growth ability than that of the wild type and OE3545 strains under MG treatment, non-optimal temperature, or 1% NaCl. The ability of DM3545 for rhizosphere colonization was lower than that of the wild-type and OE3545 strains. The DM3545 strain showed substantially reduced virulence toward tomato plants than its wild-type and OE3545 counterpart. Moreover, DM3545 was more sensitive to MG in plants than the wild-type and OE3545 strains. These results suggest that YceI is involved in the adaptability of *R. solanacearum* to the presence of MG and the effect of other tested abiotic stresses. This protein is also possibly engaged in the virulence potential of *R. solanacearum*.

## 1. Introduction

*Ralstonia solanacearum* is regarded as the most destructive plant pathogenic bacterium with a wide range of geographical distributions and hosts [1]. The worldwide economic losses caused by *R. solanacearum* on potato alone is estimated at USD 1 billion each year [2]. Given the wide range of *R. solanacearum* hosts, unique pathogenic mechanism, and an especially viable but not culturable state, plant bacterial wilt is difficult to control [3]. Chemical control is one of the effective measures to control plant bacterial wilt. The bactericides used to control plant bacterial wilt in China include chloroisobromine cyanuric acid, copper sulfate-ammonia complex and thiediazole copper, etc. [4]. However, the pathogen has developed resistance to these bactericides after long-term use [5,6,7]. Several genes can make *R. solanacearum* resistant to drugs. The *rpoS* of *R. solanacearum* is related to the resistance to copper preparations, such as thiodiazole-copper [8]. Moreover, the genes, such as intimal transporter gene *cmeB*, undecaprenyl pyrophosphate phosphate gene *bcrC*, thiopurine methyl transferase gene, and multi-drug resistance genes, are also related to the function of drug resistance [9,10,11]. Studies have shown that drug resistance in bacteria is not only related to resistance genes but also to membrane efflux pumps, persistent state, and viable but not culturable state [12,13]. Thus, new bactericides should be developed to control the disease.

More than 400,000 secondary metabolites, produced by more than 1600 plants, exhibit antibacterial activity [14]. The 3,4,5-trihydroxybenzoic acid methyl gallate (MG), which is a kind of aliphatic gallate, exists in numerous plants and possesses various biological activities [15,16,17]. MG inhibits the growth of various plant pathogens, including *R. solanacearum* Rs-T02 (phylotype I, sequevar 14) [18]. MG shows a good controlling effect on tomato bacterial wilt caused by *R. solanacearum* Rs-T02 [19], which indicates that it has the potential to be considered as a bactericide for controlling plant bacterial wilt.

The bacteriostatic mechanism of MG on *R. solanacearum* mainly occurs through its effect on the cell structure and function, energy metabolism and biosynthesis of metabolites [19]. Moreover, AVT05_RS03545 protein in *R. solanacearum* Rs-T02, which is homologous to the Rsp1238 protein in *R. solanacearum* GMI1000 (phylotype I, race l, biovar3) and predicted as polyisoprenoid-binding protein YceI, was up-regulated in *R. solanacearum* treated with MG, as revealed by two-dimensional electrophoresis [20]. Our previous transcriptome sequencing also showed that AVT05_RS03545 of Rs-T02 was up-regulated significantly under MG stress [21]. We suspected that protein YceI may be involved in the adaptability of *R. solanacearum* to MG-induced stress.

The YceI proteins are a family of bacterial lipocalins (BCNs) including base-induced periplasmic proteins and polyisoprenoid-binding protein [22]. They all have the structure of an extended, eight-stranded, and antiparallel b-barrel reminiscent of the lipocalin fold [23,24]. The YceI of *Burkholderia cenocepacia* plays essential roles in the transport of respiratory electrons, inhibition of peptide polymyxin B (PmB), and resistances to other bactericidal antibiotic [25]. YceI Hp1286 of *Helicobacter pylori*, a YceI ortholog of *B. cenocepacia*, is involved in antibiotic resistance [22].

In this article, we present the biological characteristics of the gene *yceI*, by the growth determination of strains under different conditions and the virulence assay. The results demonstrated that YceI is involved in the adaptability of *R. solanacearum* to the presence of MG and the effect of other tested abiotic stresses. This protein is also engaged in the virulence potential of *R. solanacearum*.

## 2. Materials and Methods

### 2.1. Bacterial Strains and Growth Condition

The wild-type strain, *R. solanacearum* RS-T02, was originally isolated from a tomato plant in Nanning City, Guangxi Province, China [26]. The strains were stored in sterilized water at 20 °C. The *R. solanacearum* strains were initially cu1tured on nutrient agar medium (NA: 3 g/L yeast extract, 5 g/L peptone, 10 g/L glucose, and 18 g/L agar) for 24–48 h or in NA liquid medium (28 °C, 130 r/min) for 24 h. The competent cells of *Escherichia coli* DH5α for vector construction were grown in Luria–Bertani (LB) medium at 37 °C. The concentration of antibiotics in the used medium was as follows: 25 µg/mL kanamycin (Km) and 50 µg/mL ampicillin (Amp). The semiselective agar (SMSA) medium was used to determine the bacterial population in the soil and plants [27].

### 2.2. Quantitative Real-Time (qRT-PCR) of yceI

qRT-PCR analysis was used to determine the expression levels of *yceI* under MG treatment. The growth condition of *R. solanacearum* and mRNA preparation method were referred to in a previous study [21]. The total RNA was reverse transcribed with HiScript Q Select RT SuperMix for qPCR +gDNA wiper (Vazyme, Nanjing, China). The qRT-PCR was conducted using the SYBR Green RT-PCR Kit (Takara, Kusatsu, Japan) on a qTower 2.2 system (AnalytikJena, Jena, Germany) with the specific primers 5′-GGCTCCACCAAAATCAAGCG-3′ and 5′-CCAGCGCAATGTTCAGATCG-3′. The 16s rRNA gene was used as an internal reference with the specific primers 5′-ATGCCACTAACGAAGCAGAGA-3′ and 5′-TTGCGGGACTTAACCCAAC-3′. Each sample was subjected to three independent PCRs (biological replicates), and each PCR reaction was performed in triplicate (technical replicates). The results were calculated using the 2^−ΔΔT^ method [28]. The expression of *yceI* in *R. solanacearum* not treated with MG was regarded as a standard to determine the relative expression of *yceI* in *R. solanacearum* under MG treatment.

### 2.3. Gene Deletion and Over-Expression in R. solanacearum

The *yceI* in *R. solanacearum* was deleted through consecutive homologous recombination. The two DNA fragments for the construction of the deletion mutant (named DM3545) of *yceI* were amplified with the primers listed in Table 1, and cloned into the suicide plasmid pK18mobsacB using a One Step Cloning Kit (Vazyme, Nanjing, China). The recombinant plasmid was accomplished and its authenticity was confirmed by enzyme digestion and Sanger sequencing. We transferred the recombinant plasmid into *R. solanacearum* by using electroporation. The second crossover recombined strains were obtained on the improved NA medium by using 10% sucrose instead of glucose. The DM3545 was screened from non-Km resistant mutants by polymerase chain reaction (PCR).

The open reading frame and the upstream fragment (totally 1088 bp) of the target gene were linked to the pBBR1MCS4 vector to obtain the recombinant vector by using a One Step Cloning Kit (Vazyme, Nanjing, China), and the DNA fragment was amplified with the primers listed in the Table 1. The recombinants were introduced into the wild-type *R. solanacearum* strains by electrotransformation, and OE3545 was screened on the NA plate with 50 µg/mL Amp by polymerase chain reaction (PCR).

### 2.4. Sensitivity of R. solanacearum to MG

In assaying the sensitivity to MG, *R. solanacearum* strains were cultured to the logarithmic growth phase and adjusted to the same concentration. The wild-type, DM3545, and OE3545 strains were cultured in NA medium with or without 20 µg/mL MG for suspension cultivation in three biological replicates. The growth was monitored every 4 h by using Bioscreen C automated system.

### 2.5. Sensitivity of R. solanacearum to Stresses (Non-Optimal Temperature, Non-Optimal pH and 1% NaCl)

The NA liquid medium, containing 1 × 10^8^ colony forming units (CFUs) mL^−1^ *R. solanacearum* Rs_T02 wild-type, DM3545, and OE3545 strains, was used as the inoculum in 96-well plates. The bacterial culture was then incubated at 20 °C, 24 °C, 28 °C, 34 °C and 38 °C for 48 h. Each treatment had three replicates. The growth of *R. solanacearum* at each temperature was examined by using Infinite M200 Microplate Reader.

The wild-type, DM3545, and OE3545 strains were cultured in sealed NA liquid medium with 1% NaCl for suspension cultivation (28 °C, 130 r/min) in three biological replicates. The growth of *R. solanacearum* under 1% NaCl treatment was examined by using an Infinite M200 Microplate Reader.

The NA liquid nutrient, which contained 1 × 10^8^ CFU mL^−1^ *R. solanacearum* Rs_T02 wild-type, DM3545, and OE3545 strains, was used as the inoculum in 96-well plates at 28 °C. The bacterial culture was then incubated at pH 4–9.1, which was adjusted using HCl and KOH to avoid the inhibition of growth by Na^+^ at high pH [29]. Each treatment involved three replicates. The growth of *R. solanacearum* was examined by using an Infinite M200 Microplate Reader.

### 2.6. Determining the Colonization and Virulence of R. solanacearum

In the soil-soak inoculation experiment, tomato plants Zhenlong NO.2 (Danong Horticulture Seed, Guangzhou, China) were cultivated in 5-cm pots. Plants (height 10–15 cm) were selected for inoculation. The wild-type, DM3545, or OE3545 strain was tested for virulence. A total of 30 mL bacterial suspension containing 1 × 10^8^ CFU mL^−1^ of *R. solanacearum* (Rs_T02 wild-type, DM3545, or OE3545 strain) was poured onto the soil near the roots of a tomato plant in the pot. Each treatment consisted of three replicates, and each replicate contained 32 tomato plants. The induced disease symptoms were scored daily using a disease index in the range 0–4 (0, symptomless plants; 1, 1–25% of leaves wilted; 2, 26–50% of leaves wilted; 3, 51–75% of leaves wilted; 4, 76–100% of leaves wilted or dead). Disease severity was calculated in accordance with the following formula: Disease severity = (Σ (number of diseased plants at each index × disease index)/(total plants investigated × highest disease index)) × 100%.

At 1 day post inoculation (DPI), bacteria were recovered from each of the rhizosphere soil samples inoculated by wild-type, DE3545, or OE3545 strains. Furthermore, 15 g soil, collected from the rhizosphere soil of 15 plants inoculated by wild-type, DE3545, or OE3545 strain, homogenized using glass sticks in test tubes with 15 mL of sterile distilled water. The weight of rhizosphere soil collected from each plant was 1 g. Each homogenate was serially diluted with sterile distilled water and plated on the SMSA medium [27]. Each treatment consisted of three replicates.

At 3, 6 and 9 DPI, five tomato plants of each treatment (plants inoculated by wild-type, DE3545, or OE3545 strains) were rinsed with sterile distilled water and sampled by cutting 2-cm-length roots and 2-cm-length mid-stems. Populations of wild-type, DE3545, and OE3545 strains in the samples were determined using the procedures described by Takeaki et al. [30] and Dai et al. [31]. Samples were weighed separately and homogenized using glass sticks in test tubes with 1 mL of sterile distilled water. Each homogenate was serially diluted with sterile distilled water and plated on the SMSA medium [27]. The plates were incubated at 28 °C for 2 days. The CFUs of bacteria recovered from plant tissues in the samples were counted. Each treatment consisted of three replicates.

### 2.7. Determining the Control Effect of MG against the Strains

The seedling inoculation method [32] was used to determine the wilting rate of plants and the bacterial colonization in roots and stems of tomato. The seedlings were treated with 20 µg/mL MG for 24 h as the treatment group (non-MG-treated seedlings were used as the control group). Then the roots of tomato plants were rinsed with sterile distilled water to avoid the MG-induced effects on the strains in vitro. The roots of each seedling were then dipped in the different bacterial inoculum (10^9^ CFU/mL of wild-type, DM3545, or OE3545 strains). Populations of wild-type, DE3545, and OE3545 strains in samples were determined using the procedures described by Takeaki et al. [30] and Dai et al. [31]. At 9 DPI, 15 tomato plants were each rinsed with sterile distilled water, and sampled by cutting 2-cm-length roots, and 2-cm-length mid-stems. The samples were weighed separately and homogenized using glass sticks in test tubes with 1 mL of sterile distilled water. Each homogenate was serially diluted with sterile distilled water and plated on the SMSA medium [27]. The plates were incubated at 28 °C for 2 days. The CFUs of bacteria recovered from plant tissues in the samples were counted. Each treatment consisted of three replicates.

The seedling inoculation method was also used to determine the control effect of MG against wild-type, DM3545, and OE3545 strains [32]. The method of seedling treatment was as described above. Each treatment consisted of three replicates; each replicate contained 28 tomato plants. The control effect of MG against the different strains (wild-type, DM3545, or OE3545) was calculated using the following formula:

Control efficiency (%) = (A − B) × 100/A

where A represents “% of wilted non-MG-treated seedlings,” and B denotes “% of wilted MG-treated seedlings.”

### 2.8. Statistical Analysis

Data were subjected to analysis of variance in SAS version 6.08. Mean comparisons were conducted via a Fisher’s least significant difference (LSD) test at *p* = 0.05. Percentage values were transformed to arcsine √% for statistical analysis. Standard error and LSD results were recorded.

## 3. Results

### 3.1. YceI Is Involved in the Sensitivity of R. solanacearum to MG

The *AVT05_RS03545* *yceI* of *R. solanacearum* was up-regulated by MG treatment, as revealed by two-dimensional electrophoresis and RNA sequencing [20,21]. In this study, qRT-PCR confirmed that the *yceI* transcription level was higher in MG-treated *R. solanacearum* than in non-MG-treated *R. solanacearum* (fold change: 51 ± 3.2).

**Figure 1:**
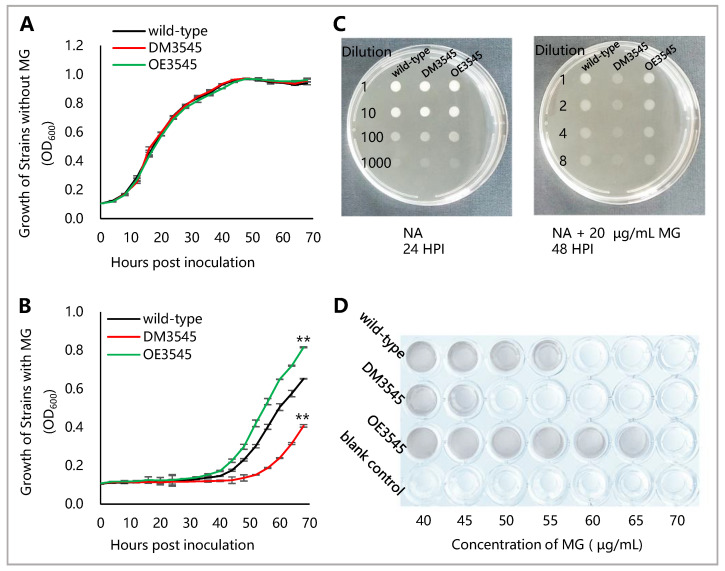
YceI is important for growth adaptability to MG in *R. solanacearum*. (**A**) Growth of the strains in NA liquid medium. (**B**) Growth of the strains in NA liquid medium with 20 µg/mL MG. Growth differences between mutants and the wild-type strain at 68 h post inoculation (HPI) are marked with asterisks, indicating *p* < 0.01 (**), which was compared with wild-type and statistically analyzed by ANOVA, based on Fisher’s LSD test. (**C**) Gradient diluted bacterial cultures were inoculated on NA medium with or without 20 µg/mL of MG. The growth of *R. solanacearum* strains was observed and photographed at 24 or 48 HPI. (**D**) Minimum bactericidal concentration (MBC) of MG against *R. solanacearum*. The wells of blank control were uninoculated NA liquid medium. After 96 h of incubation in 96-well plates, the wells that were free of turbidity indicated that the stains were inhibited by a special concentration of MG.

The deletion mutant (DM3545) and over-expression strain (OE3545) were constructed to further study the role of YceI in the adaptability of MG. The growth of the strains in the NA liquid medium was detected to study the relationship between YceI and the sensitivity of *R. solanacearum* to MG. The results showed that, in NA liquid or on solid medium without MG, the growth level was similar among all of the different strains (Figure 1A,C), indicating that the YceI was not involved in bacterial survival under optimal condition. However, in NA liquid or on solid medium with 20 µg/mL MG, OE3545 and wild-type strains exhibited better than DM3545 (Figure 1B,C). Consistent with the above results, the minimum bactericidal concentration (MBC) of MG against OE3545 strain (MBC: 70 µg/mL) and wild-type (MBC: 60 µg/mL) were higher than that of MG against the DM3545 strain (MBC: 50 µg/mL) (Figure 1D). Moreover, the over-expression of YceI improved the tolerance of *R. solanacearum* to MG. The results indicated that YceI is important for growth adaptability to MG in *R. solanacearum*.

### 3.2. YceI Is Involved in Adaptability of R. solanacearum to Stresses including Low Temperature, and 1% NaCl but Not to Non-Optimal pH

**Figure 2:**
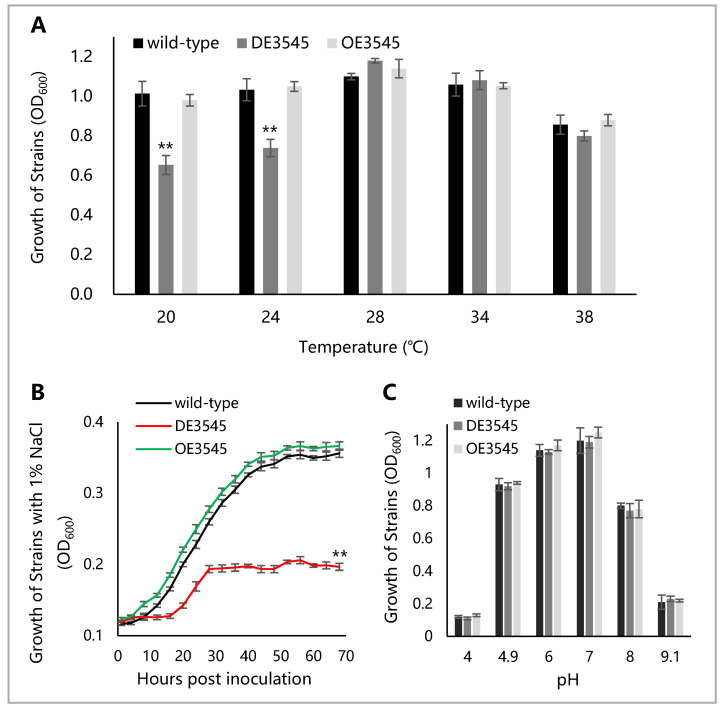
Growth of *R.* *solanacearum* under non-optimal temperature, 1% NaCl, and non-optimal pH treatment. The figure marked with asterisks, indicating *p* < 0.01 (**), was compared with the wild-type and statistically analyzed by ANOVA, based on Fisher’s LSD test. (**A**) Growth of three strains in NA liquid medium under given temperature (coordinate-axis X) at 48 HPI. (**B**) Growth of strains in NA liquid medium with 1% NaCl. (**C**) Growth of strains in NA of various pH at 48 HPI.

YceI is involved in cellular stress response in *Helicobacter pylori* and *Escherichia coli* [33,34,35,36]. The sensitivity of wild-type and mutant strains to temperature, NaCl and pH was tested to verify the adaptability of YceI in *R. solanacearum* to stress. The results showed that the growth of DM3545 was attenuated in NA liquid medium at 20 °C and 24 °C (Figure 2A), indicating that YceI is involved in growth adaptability to low temperature stress.

The growth level is similar between wild-type and OE3545 strains under 1% NaCl stress (Figure 2B). The DM3545 strain showed attenuated growth in the medium under 1% NaCl stress (Figure 2B), indicating that YceI is involved in the growth adaptability to NaCl stress.

The growth of *R. solanacearum* in NA liquid medium with different pH at 48 HPI showed that the growth of all of the strains was without a significant difference under pH-stress (Figure 2C), indicating that the YceI is not related to the adaptability of *R. solanacearum* to pH stress.

### 3.3. YceI Is Associated with Colonization and Virulence of R. solanacearum

**Figure 3:**
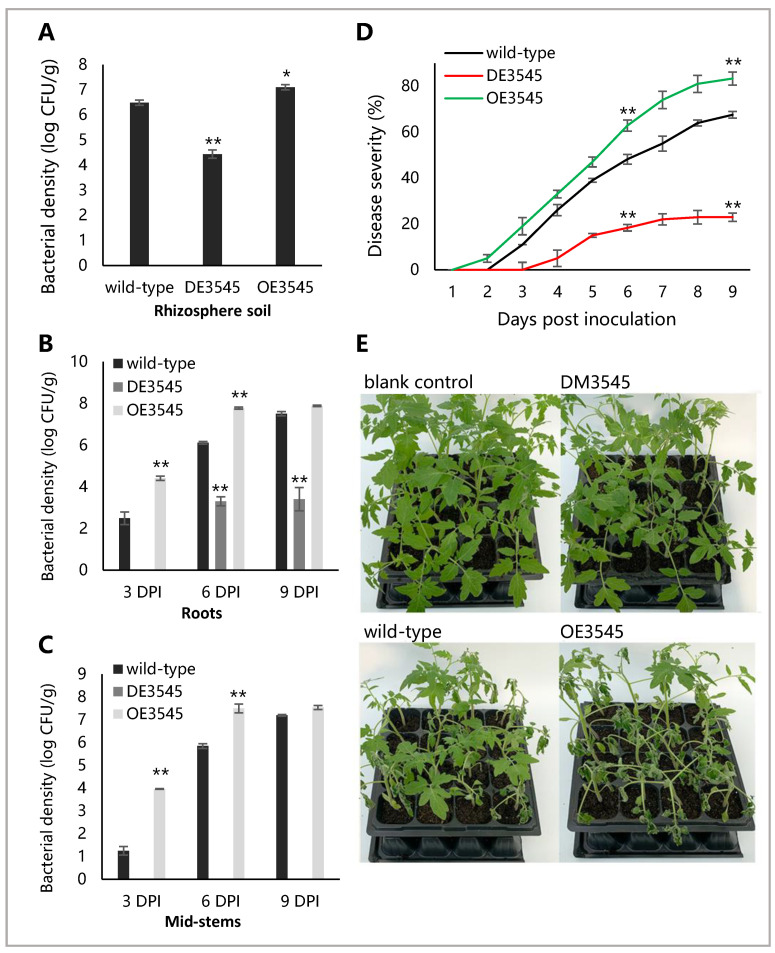
Determining the bacterial population in the soil and plants by soil-soak inoculation method. The figure marked with asterisks, indicating *p* < 0.01 (**) or *p* < 0.05 (*), was compared with the wild-type and statistically analyzed by ANOVA based on Fisher’s LSD test. The bacterial population (CFU/g of fresh weight) was log-transformed. (**A**) Amount of bacterial CFUs per gram of soil tissue at 1 DPI. (**B**) Amount of bacterial colony forming units per gram of roots tissue at 3, 6 and 9 DPI. (**C**) The amount of bacterial CFUs per gram of mid-stems tissue at 3, 6 and 9 DPI. No bacterial colony of DE3545 was detected. (**D**) Disease severity of three strains at 1–9 DPI. (**E**) Growth of *R. solanacearum*-inoculated tomato plants at 9 DPI.

To investigate the effect of the reduced stress-adaptability of DM3545 on its virulence to tomato plants, we inoculated the sensitive tomato plants with wild-type, DM3545, and OE3545 strains by soil-soak inoculation. In Figure 3A, the number of CFUs of OE3545 and wild-type strains in rhizosphere soil were higher than that of DM3545 (*p* < 0.01), whereas the number of CFUs of OE3545 strain was higher than that of the wild-type (*p* < 0.05), indicating that YceI is important for the colonization ability of *R. Solanacearum* in tomato rhizosphere soil.

At 9 DPI, the bacterial populations of the DM3545 strains was significantly lower than that of wild type and OE3545 strains in roots and mid-stems (Figure 3B,C), indicating that the colonization ability of DM3545 was significantly lower than that of wild type and OE3545 strains (*p* < 0.01), suggesting that the YceI is involved in the colonization ability of *R.* *solanacearum* in the roots and stems of tomato.

Disease severity of OE3545 and wild-type strains was higher than that of DE3545 strain (Figure 3D), and OE3545 strain showed a better disease severity than the wild-type (*p* < 0.01). At 3 DPI, the plants inoculated with wild-type and OE3545 strains showed symptoms of leaf wilting. At 9 DPI, most of the plants inoculated with wild-type and OE3545 strains wilted. However, most of the plants inoculated with DM3545 strain were healthy (Figure 3E). As the YceI probably has a role in general bacterial survival, the results above suggested that YceI may be indirectly involved in the virulence of *R.* *solanacearum* to tomato.

### 3.4. Control Effect of MG on Bacterial Wilt Caused by yceI-Deletion Mutant

**Figure 4:**
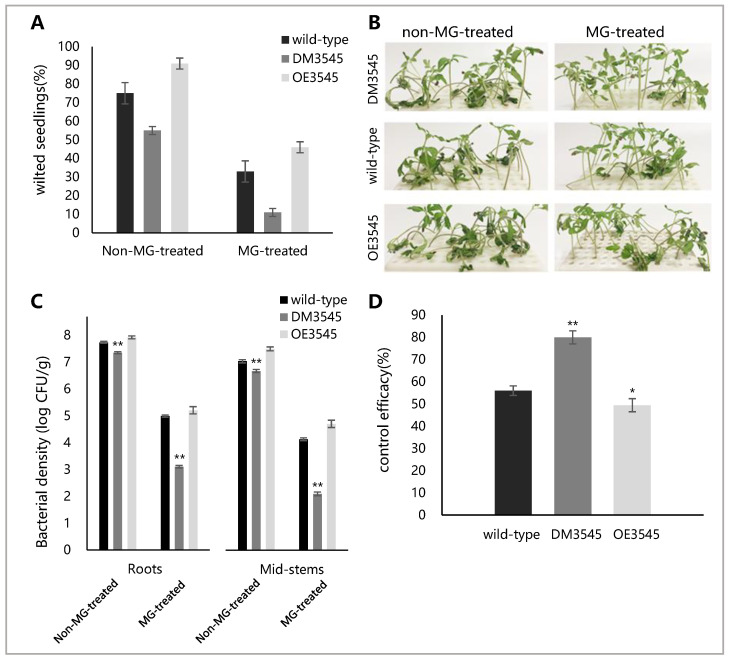
Determining the control effect of MG on bacterial wilt by seedling inoculation method. The figure marked with asterisks, indicating *p* < 0.01 (**) or *p* < 0.05 (*), was compared with the wild-type and statistically analyzed by analysis of variance (ANOVA) based on Fisher’s LSD test. (**A**) Wilting rate of plants at 9 DPI. (**B**) Growth of *R. solanacearum*-inoculated tomato seedlings at 9 DPI. (**C**) Bacterial population in roots and mid-stems at 9 DPI. The bacterial population (CFU/g of fresh weight) was log-transformed. (**D**) Control effect of MG against wild-type, DM3545 or OE3545 strain at 9 DPI.

Seedling inoculation was used to determine the role of YceI in the control effect of MG on bacterial wilt. The tomato seedlings were treated with 20 µg/mL MG for 24 h as the treatment group before inoculation (non-MG-treated seedlings as the control group). The wilting rate and bacterial population were measured at 9 DPI. The wilting rates of the plants untreated with MG were higher than that of the plants treated with MG (Figure 4A). Most of the plants untreated with MG and inoculated with wild-type and OE3545 strains wilted (Figure 4B). However, most of the plants treated with MG and inoculated with DM3545 strain were healthy (Figure 4B). In Figure 4C, the density of the DM3545 bacterial culture was lower than those of the other two strains in roots and mid-stems, and the bacterial populations in plants untreated with MG were higher than that in plants treated with MG, verifying the validity of the plant wilting rate in Figure 4A.

The data of wilting rate were used to calculate the control effect. The control effect of MG against the DM3545 strain (80.15%) was higher than that of MG against the wild-type (56.45%) or OE3545 strain (49.36%) (Figure 4D), indicating that the *yceI*-deletion strain was more sensitive to MG in plants.

## 4. Discussion

The YceI proteins are a family of BCNs [22]. Lipocalins are best known for their binding of a remarkable array of small hydrophobic ligands. The cavities of YceI can bind to amphiphilic ligand molecules, including PmB and ubiquinone-8 [25,37]. The protein encoded by AVT05_RS03545 in *R. solanacearum* was predicted as a YceI family protein. YceI-family proteins may act by sequestering chemicals within the periplasmic space, thus alleviating chemical toxicity to a certain degree. El-Halfawy and Valvano [25] observed that mutants with a double deletion of BCAL3310 and BCAL3311 encoding the two YceI homologues in *Burkholderia cenocepacia* had increased sensitivity to the antimicrobial peptide PmB. Complementing the double-deletion mutant with both genes restored the resistance to PmB to the parental level. Purified YceI BCAL3310 and BCAL3311 can bind the PmB-Oregon green 514 conjugate, which sequestered PmB-protected cells from the toxic effects of the antibiotic [25]. In this article, we discovered that YceI is involved in the growth ability of the wild-type, DM3545, and OE3545 strains under the treatment of MG and 1% NaCl. We speculate that YceI can probably sequester MG within the periplasmic space to prevent MG from harming *R. solanacearum*. However, we also have not determined whether YceI in *R. solanacearum* can bind to NaCl, which needs to be confirmed by future research.

The pH of the environment is one of the factors affecting the survival of microorganisms. Microorganisms can adapt to the pH of the environment through physiological and biochemical activities. Polyprenyl pyrophosphate-binding protein Hp1286 from *Helicobacter pylori* is a YceI family protein and plays a relevant role in bacterial colonization and persistence in the stomach [34,35,36]. YceI Hp1286, which has the binding specificity of amphiphilic compounds with a linear chain of about 22 carbon atoms, is involved in adaptation to an acidic environment. The function of YceI Hp1286 can be the sequestering of specific fatty acids from the environment [34]. Stancik [23] observed that the periplasmic protein YceI was induced under high pH in *Escherichia coli*, but its functions have not been characterized. Our study showed no correlation between YceI and the growths of the wild-type, DM3545, and OE3545 strains in the absence of specific fatty acids under pH stress. Whether YceI of *R. solanacearum* is tolerant to specific fatty acids needs further study.

YceI-family proteins with a multitude of functions in microorganisms are diverse. In addition to sequestering chemicals and persistence in acid stress [35], YceI-family proteins from *Thermus thermophilus* HB8 function in isoprenoid transport or storage protein and may serve as a part of an unknown isoprenoid metabolic pathway [24]. *E. coli* harbors a periplasmic YceI protein that responds to alkaline stress, but its function has not otherwise been characterized [23]. We observed that YceI in *R. solanacearum* is involved in the adaptation under the treatment of low temperature. Changes in the lipid composition of certain bacteria are associated with the cellular response to temperature stresses [38]. YceI may bind to specific fatty acids [35], implying that YceI, probably as a lipid-transport protein, is involved in cellular stress response.

MG inhibited the growth of *R. solanacearum* and reduced the incidence rate of tomato bacterial wilt [18]. In this study, the deletion of *yceI* significantly reduced the resistance of *R. solanacearum* to MG and the virulence on tomato. After the strains were poured onto the soil near the roots of the tomato plant in the pot. No bacterial colony of *yceI*-deletion mutant has been detected in mid-stems at 3, 6 and 9 DPI. The mechanism of limitation of the bacterial multiplication in the stem needs further study. Additionally, it would be interesting to look further than 14 days to see whether the bacterial multiplication in the stems was completely suppressed in plants.

Meanwhile, the population of the *yceI*-deletion mutant in tomato and rhizosphere soil was lower than that of wild-type and over-expression strains. However, the growth of the *yceI*-deletion mutant was the same as that of wild-type and over-expression strains in the NA medium, indicating that specific factors in tomato rhizosphere soil affected the survival of the *yceI*-deletion mutant. The decrease in virulence may be related to the reduced population of the *yceI*-deletion mutant. The specific factors affecting the survival of the *yceI*-deletion mutant need to be further studied. The rhizosphere soil and plant host induce various stresses, such as pH, salicylic acid, lignin, and phenols, which can inhibit the growth of *R. solanacearum* [39,40,41,42]. The decrease in reproduction and virulence of the deletion mutant in host may be due to the decrease in stress tolerance.

In conclusion, there is a positive relationship between YceI and the growth of the strains under the treatment of MG, low temperature, or 1% NaCl. In the soil-soak inoculation experiment, the pathogenicity toward tomato plants of wild-type strain was stronger than the DM3545. In addition, the seedling inoculation experiment suggested that YceI is involved in the higher sensitivity of DM3545 strain to MG than that of wild-type and OE3545 in plants. These results suggest that YceI is involved in the adaptability of *R. solanacearum* to the presence of MG and the effect of other tested abiotic stresses. This protein is also possibly engaged in the virulence potential of *R. solanacearum.*

## Figures and Tables

**Table 1:** Primers used for construction of deletion mutant and over-expression strain in this study.

Primer	Sequence/5′-3′	
Left-arm1	CGACGGCCAGTGCCAAGCTTATTGCCGGAATCAGGGTGTC	For deletion mutant
Left-arm2	AAGTTCGAGACCCAGCTCAACGCTGGAGCTGCTCAAGAAG	For deletion mutant
Right-arm1	CTTCTTGAGCAGCTCCAGCGTTGAGCTGGGTCTCGAACTT	For deletion mutant
Right-arm2	ATGACCATGATTACGAATTCGATTCGGTCAGCGCATAGCC	For deletion mutant
ORF1	TCACACAGGAAACACATATGGGTGAAGCGGATCGTGCCGG	For over-expression strain
ORF2	TCGATACCGTCGACCTCGAGTCAGGGCTTCTTGAGCAGCT	For over-expression strain

## Data Availability

Not applicable.
